# Online versus in-person Eating Disorder Examination for adolescents with eating disorders: Empirical verification of data equivalency

**DOI:** 10.1192/j.eurpsy.2023.2200

**Published:** 2023-07-19

**Authors:** N. Besse-Fluetsch, L. Mayr, L. Smigielski, C. Buehlmann, D. Pauli

**Affiliations:** 1 Department of Child and Adolescent Psychiatry ans Psychotherapy; 2 Psychiatric University Hospital Zürich, Zürich, Switzerland

## Abstract

**Introduction:**

In the last ten to fifteen years, it has become common for researchers to collect both quantitative (Sue & Ritter, 2012) and qualitative data (Jowett, Peel, & Shaw, 2011) online. The Covid-19 pandemic has increased the importance of this process and accelerated it in many disciplines (Torrentira, 2022).

In addition to convenience, recent work suggests that online data collection may be more valid than face-to-face data collection for some populations. This would mean that online data collection may be the most valid and effective for this age group (Barratt, 2012).

**Objectives:**

Adolescents with an eating disorder tend to be more open about their symptoms via impersonal online data collection than they are in a face-to-face conversation. Symptom underrating has been documented in face-to-face interviews, because “of feelings of shame elicited by the loss of anonymity during face to-face interviews” leading to face-to-face denial, whereas a self-report questionnaire allows for more privacy and hence honesty while answering questions (Berg et al. 2011). This is especially key in the diagnosis of Anorexia Nervosa (AN), as AN patients minimize, deny, and/or fail to recognize their symptoms (Passi, Bryson and Lock 2003).

Given the benefits of collecting data online for both researchers and participants, it is important to determine the quality of the data collected online to guide its use and interpretation. More evidence is needed to confirm the equivalence of online and face-to-face interview data. The current study examines the equivalence of semi-structured interview data collected online versus original face-to-face interviews.

**Methods:**

The Eating Disorder Examination (EDE), assessing psychopathology of eating disorders, was administered to 49 adolescents meeting ICD 10 criteria for anorexia nervosa or atypical anorexia nervosa. The same diagnostic interview was administered twice, once via face-to-face and once as an online version, within a week. Method order was counterbalanced among participants and temporal stability was controlled. The Eating Disorder Inventory-2 (EDI-2) was used as a control variable.

**Results:**

Both the equivalence test and the null hypothesis test were significant for the sum score of the EDE. Measures of psychopathology in eating disorders demonstrated equivalence across face-to-face and online format of the EDE.

**Conclusions:**

The aim was to examine the equivalence of face-to-face and online methodologies, controlling for temporal change in the variable under investigation over one week and order of administration. Results demonstrate equivalence across face-to-face and online format of the EDE. These findings suggest that data obtained using EDE online can be interpreted in comparison with normative data obtained in the face-to-face Interview and that corrections trough transformations are not necessary.

**Disclosure of Interest:**

None Declared

